# Free Thyroxine as a Predictor of Mortality in Critically Ill Septic Patients—A Retrospective Study

**DOI:** 10.3390/diagnostics16050680

**Published:** 2026-02-26

**Authors:** Matei Florin Negruț, Vlad Pastor, Robert Bolcaș, Oana Antal, Robert Szabo, Cristina Petrișor

**Affiliations:** 1 Faculty of Medicine, “Iuliu Hațieganu” University of Medicine and Pharmacy, 400012 Cluj-Napoca, Romania; vlad_pastor@yahoo.com; 2 Anesthesia and Intensive Care II Department, “Iuliu Hațieganu” University of Medicine and Pharmacy, 400012 Cluj-Napoca, Romania; robert_bolcas@yahoo.com (R.B.); antal.oanna@gmail.com (O.A.); szabo.robert@elearn.umfcluj.ro (R.S.); petrisor.cristina@umfcluj.ro (C.P.)

**Keywords:** euthyroid sick syndrome (ESS), non-thyroidal illness syndrome (NTIS), sepsis, septic shock, ICU mortality, free thyroxine (fT4), prognostic biomarkers

## Abstract

**Background/Objectives:** Euthyroid sick syndrome (ESS), and particularly low T3, have been associated with increased mortality in septic patients, yet the prognostic value of free thyroxine (fT4) remains controversial. This study aims to evaluate the association between fT4 on ICU admission and mortality in septic patients. **Methods:** We conducted a single-center, retrospective observational study including 149 adult patients with sepsis or septic shock admitted to the Anesthesia and Intensive Care I Department of the Cluj County Emergency Hospital, Cluj-Napoca, Romania, between January 2019 and September 2025. Free T4 and thyroid-stimulating hormone (TSH) levels were measured within 24 h of ICU admission. The primary outcome was 28-day mortality, and the secondary outcome was in-hospital mortality. Demographic data, comorbidities, severity scores (SOFA, APACHE II), laboratory parameters, and outcomes were analyzed. Univariate and multivariate logistic regression analyses were performed, and predictive performance was assessed using area under the receiver operating characteristic curve (AUROC). **Results:** A total of 149 patients were included. Twenty-eight-day mortality was 29.73%, and 53.57% in patients with sepsis and septic shock, respectively. Serum fT4 was significantly lower in non-survivors, for both primary and secondary outcome (*p* = 0.01 and *p* = 0.014, respectively), whereas TSH levels were similar between groups. In the univariate analysis, fT4 showed moderate predictive ability for mortality (AUROC 0.615 and 0.632). Multivariate models, including age, hemoglobin, SOFA score, and fT4, showed a greater discriminative performance (AUROC 0.805 and 0.799). **Conclusions:** Lower fT4 levels on ICU admission seem to be independently associated with increased mortality in septic patients. Incorporating fT4 into multiparametric prognostic models might improve early risk stratification in sepsis, particularly in settings where other thyroid parameters are not routinely available.

## 1. Introduction

Sepsis and septic shock are a leading cause of preventable mortality among critically ill patients worldwide, accounting for around 19.7% of all deaths annually [1,2]. According to the Sepsis-3 criteria, sepsis is defined as a life-threatening organ dysfunction caused by a dysregulated host response to infection, while septic shock is defined by vasopressor requirements for maintaining mean arterial pressure (MAP) above 65 mmHg, or lactate levels ≥ 2 mmol/L, despite adequate fluid resuscitation [3].

Similar to global trends, recent data indicate a progressive increase in sepsis rates within Romanian medical centers, rising from 0.26% in 2007 to 2.85% in 2024 [4]. This upward trend is associated with rising costs in treatment and increased burden on the global healthcare system [5,6]. Clinical risk stratification tools could guide treatment, resource allocation, and improve patient outcomes [7].

Alterations in thyroid hormone levels have been extensively documented in critically ill patients, leading to euthyroid sick syndrome (ESS) definitions, also known as non-thyroidal illness syndrome (NTIS) or low T3 syndrome. Features of ESS include low levels of T3, normal or low levels of T4, increased plasma rT3, and normal or low TSH [8,9,10,11]. Increased mortality of patients with ESS has been reported in multiple publications, both for general intensive care unit (ICU) patients [12,13,14], and specifically for septic patients [7,11,15,16,17,18].

While changes in thyroid hormone levels may be adaptive in the early stages of critical illness, leading to decreased energy requirements and decreased protein catabolism, during the chronic phase, these changes may become maladaptive, further exacerbating sepsis-induced cardiomyopathy, immunosuppression and respiratory failure [11,19,20]. Moreover, severely decreased T3 levels were associated with an increased prevalence of persistent inflammation, immunosuppression, and catabolism syndrome (PICS) in sepsis [21] patients.

Therefore, ESS is a complex and dynamic spectrum of biochemical alterations [9]. Initially, serum T3 levels drop due to reduced peripheral conversion of T4 to T3 and altered deiodinase activity, thus leading to increased levels of rT3 and normal or increased levels of T4 [9,13,22]. Later on, T4 and fT4 levels start to decrease as well [9]. Therefore, two clinically distinct stages of ESS can be defined: a “low T3 syndrome” and a “low T3T4 syndrome” [20,23]. During the chronic phase, TSH levels can also decrease due to central hypothalamus-pituitary-thyroid (HPT)-axis inhibition by circulating inflammatory mediators [9,10,15].

Although decreased levels of thyroid hormones have been repeatedly reported in septic patients over the past few decades, due to the heterogeneity of patient populations, study design and the fraction of hormones investigated (free vs. total T3 or T4), no consensus has been reached regarding the predictive power of thyroid hormones for mortality. Most studies have correlated low T3 and mortality, with varying results regarding fT3, total T4 and fT4 [11].

A recent study, including 888 critically ill patients, reported a biphasic response between fT4 and mortality: both fT4 < 1.2 μg/dL and fT4 between 1.9 and 3 μg/dL were associated with increased mortality, when compared to patients with fT4 outside of these ranges [8]. While fT3 was shown to have a superior predictive power for mortality compared to fT4 in some studies [7], other studies reported no difference in fT3 levels between survivors and non-survivors, while fT4 was significantly lower amongst non-survivors [17].

The prognostic relevance of thyroid-stimulating hormone (TSH) levels in sepsis remains controversial. While several studies report no significant association between TSH and clinical outcomes [7,15,16,17,18], others have demonstrated that lower TSH values are linked to increased mortality [24].

Currently, data on thyroid hormone profiles and sepsis mortality in Eastern Europe is limited. In Romania, serum fT4 levels are routinely measured (as opposed to fT3, total T3 or T4). As this parameter is systematically available in Romania, we aimed to investigate the predictive ability of fT4 levels on mortality in critically ill septic patients in a Romanian ICU.

## 2. Materials and Methods

We conducted a single-center, retrospective study, including septic patients admitted to the Anesthesia and Intensive Care I Department of the Cluj County Emergency Hospital, Cluj-Napoca, Romania, between January 2019 and September 2025. In this STROBE-compliant, observational study, we included patients with sepsis and septic shock, as defined by Sepsis-3 criteria, in whom tests for thyroid function were performed.

The exclusion criteria were age < 18 years old; thyroid disease documented prior to ICU admission; hormone replacement therapy within the previous 6 months (except for insulin); chronic glucocorticoid therapy or dopamine administration during ICU admission; pregnancy or childbirth within the previous six months; and lack of data regarding fT4 levels.

We collected data from the hospital’s electronic records, as follows: age and sex of the patients; SOFA and APACHE II scores within the first 24 h; underlying pathology (medical vs. surgical); type of admission (scheduled vs. emergency); ICU admission diagnosis (sepsis vs. septic shock); source of sepsis; presence of comorbidities; laboratory on admission (fT4, TSH, C-reactive protein, procalcitonin, leukocytes, platelets, hemoglobin, creatinine); ICU length of stay (LOS ICU); total hospital length of stay (total LOS); and mortality. Free thyroxine and TSH were measured using electrochemiluminescence immunoassay (ECLIA).

The primary outcome was 28-day mortality (starting from ICU admission), and the secondary outcome was in-hospital mortality (throughout the entire hospitalization). Patients were divided into “survivor” and “non-survivor” groups, according to both 28-day mortality and total in-hospital mortality, in order to analyze potential prognostic variables for the primary and secondary outcomes.

The protocol for this retrospective study was approved by the Ethics Committee of the Cluj-Napoca County Emergency Hospital (Approval No. 365/09.01.2025) for the entire study period. Our research was conducted in accordance with the Declaration of Helsinki.

Statistical analysis was performed using SPSS Statistics v27.0.0 (IBM Corp., Armonk, New York, NY, USA) and GraphPad Prism 8. Quantitative variables were reported as mean ± standard deviation and compared using Student’s *t*-test, or reported as median (quartile 1; quartile 3) and compared using the Mann–Whitney U test, as appropriate. Normality of data was assessed using the Shapiro–Wilk test. When comparing quantitative data across multiple groups, ANOVA for independent samples or the Kruskal–Wallis tests were used. Qualitative variables were reported as frequency and percentages and compared using Chi-square or Fisher’s exact test.

Univariate binary logistic regressions were performed to analyze potential predictors of mortality. Variables shown to be independent risk factors for mortality were included in a multivariate logistic regression. The predictive power of each variable and of the multivariate models were assessed using the area under the ROC curve (AUROC). For each variable, the odds ratio (OR) and 95% confidence interval (CI) were provided. We used a significance threshold of *p* < 0.05.

## 3. Results

### 3.1. Study Population

**Figure 1:**
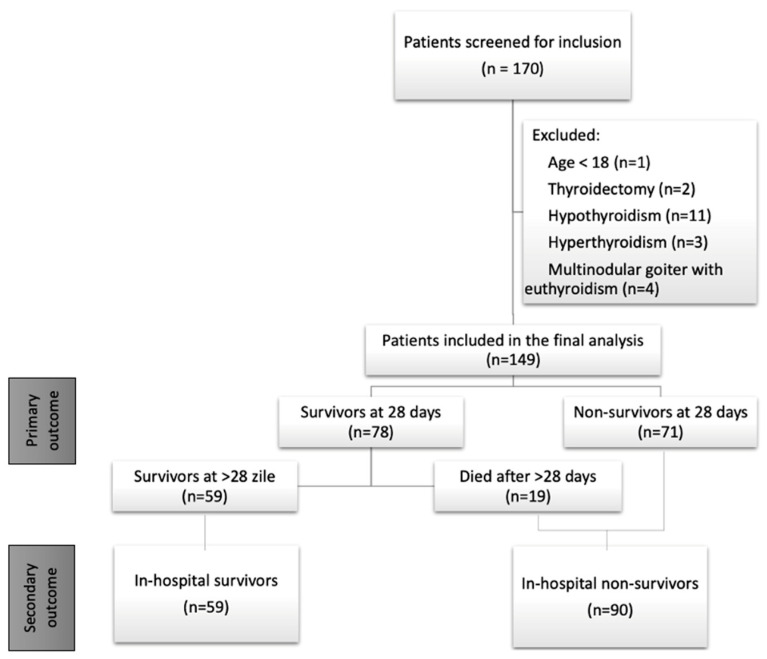
STROBE flowchart of patient selection and group allocation.

During the study period, 1458 patients with sepsis and septic shock were admitted to our ICU. Of those, only 170 patients had their fT4 and TSH levels measured. We excluded 21 patients, 20 of which had prior thyroid pathologies, and one patient being <18 years old (Figure 1).

Twenty-eight-day mortality was 47.65%, and in-hospital mortality was 60.4% in our study population. Out of the 149 patients, 112 (75.17%) had a primary diagnosis of septic shock, while 37 (24.83%) had sepsis without shock on ICU admission. In the sepsis group, 28-day mortality was 29.73%, and 53.57% in the septic shock group. The most common sources of infection were pulmonary and digestive, each accounting for around 40% of all patients. A higher median age and greater prevalence of septic shock (as opposed to sepsis without shock) were observed among non-survivors (both for 28-day and in-hospital mortality), while patients admitted with a primary medical diagnosis (as opposed to surgical pathology) had a higher 28-day mortality, but not in-hospital mortality (Table 1).

### 3.2. Analysis of Comorbidities and Clinical Outcome Scores

A summary of patients’ comorbidities, APACHE II and SOFA scores on ICU admission and LOS is provided in Table 2. While congestive heart failure, chronic kidney disease and chronic pulmonary obstructive disease were all associated with higher 28-day mortality, congestive heart failure was the only comorbidity associated with higher in-hospital mortality in our patient population. APACHE II and SOFA scores were higher among non-survivors, for both primary and secondary outcomes. While survivors and non-survivors had similar ICU and hospital LOS when grouped by in-hospital mortality, non-survivors had significantly shorter ICU and hospital LOS when accounting for 28-day mortality.

### 3.3. Analysis of Laboratory Parameters

A summary of patients’ laboratory parameters, measured within 24 h of ICU admission, is provided in Table 3. Leukocyte count was significantly higher in non-survivors when patients were grouped by in-hospital mortality, but not in the 28-day mortality groups. Hemoglobin levels were lower and serum creatinine was higher among non-survivors (for both primary and secondary outcome), while TSH levels were similar between the study groups. Serum fT4 was significantly lower in non-survivors (1 ± 0.35 vs. 1.15 ± 0.34 ng/dL, *p* = 0.01, for 28-day mortality; 1.02 ± 0.37 vs. 1.16 ± 0.31, *p* = 0.014, for in-hospital mortality).

### 3.4. Analysis of Patients Grouped by fT4 Levels

Patients were further divided into three groups, based on their fT4 levels on ICU admission. Most of the patients had fT4 values within the normal range of 0.92–1.68 ng/dL (92 patients, 61.74%), and were included in the “Euthyroid” group, while 50 (33.56%) patients had fT4 < 0.92 ng/dL (“Hypothyroid” group) and 7 (4.7%) had fT4 > 1.68 ng/dL (“Hyperthyroid” group). APACHE II SOFA scores and LOS were then compared between these three groups, and no significant difference was found between them. In-hospital and 28-day mortality was different between the three groups, with hypothyroid patients having the worst prognosis (Table 4). However, given the limited number of patients in the “Hyperthyroid” group (*n* = 7), no clinical conclusion can be drawn regarding this group.

Patients in the “Hypothyroid” group were then further divided based on TSH levels, as following: those with normal or elevated TSH levels were included in the “concordant TSH” group (*n* = 43), while those with low TSH were included in the “discordant TSH” group (*n* = 7). The normal range for TSH in the hospital’s laboratory is 0.27–4.2 μUI/mL. Severity scores, LOS and mortality were similar between the two groups (Table 5).

### 3.5. Univariate Analysis

Results of univariate binary logistic regressions for 28-day and in-hospital mortality are summarized in Table 6. All potential prognostic factors that we investigated were associated with in-hospital mortality, while serum creatinine was the only variable that did not reach statistical significance for 28-day mortality. AUROC varied between 0.308 (no predictive power) and 0.727 (good predictive power). Absolute fT4 values have a moderate predictive power (AUROC = 0.615 for 28-day mortality and 0.632 for in-hospital mortality), while hypothyroid status (as opposed to euthyroidism), defined as fT4 < 0.92 ng/dL, is moderately associated with mortality (AUROC = 0.545 for 28-day mortality and 0.596 for in-hospital mortality). The SOFA and APACHE II score had similar predictive abilities for both primary and secondary outcomes.

### 3.6. Multivariate Analysis

**Figure 2:**
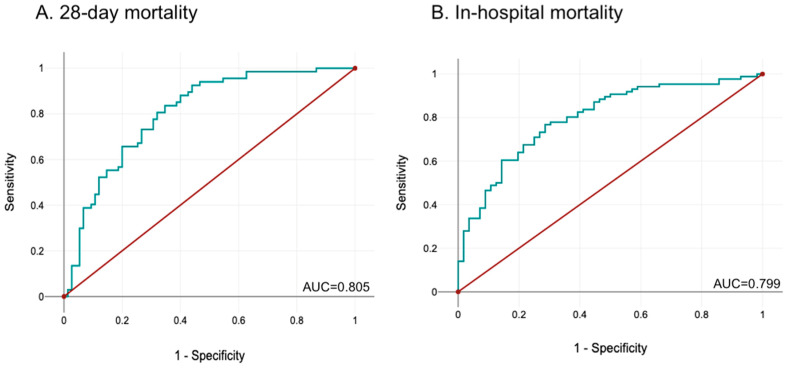
ROC curves for the multiparametric models for prediction of: (**A**) 28-day mortality; (**B**) in-hospital mortality.

A multivariate regression analysis was also performed, including age, hemoglobin levels, fT4 and SOFA score upon ICU admission (Table 7). The multiparametric models showed a very good predictive ability for 28-day mortality (AUROC = 0.805, 95% CI = 0.733–0.876, *p* < 0.001) (Figure 2A) and a good predictive ability for in-hospital mortality (AUC = 0.799, 95% CI = 0.725–0.873, *p* < 0.001) (Figure 2B).

## 4. Discussion

We investigated the potential association between fT4 and TSH levels and all-cause mortality in critically ill patients diagnosed with sepsis or septic shock by Sepsis-3 criteria.

In a meta-analysis conducted by Bauer et al. [6], including 170 studies, average mortality was 24.4% (95% CI 21.5–27.2%) for sepsis, and 34.7% (95% CI 32.6–36.9%) for septic shock, ranging from 15% to 56% between studies. The higher mortality observed in our study may be attributed to the advanced age, high APACHE II and SOFA scores, and multiple comorbidities in our patient population. Moreover, with our center being a tertiary hospital, most severe cases from the entire north-west region of the country are centralized here. In addition, the study period encompassed the COVID-19 pandemic, during which an increased mortality in septic patients was recorded globally due to viral and bacterial co-infection, limited access to healthcare services and delayed diagnosis and treatment [25,26].

In our cohort, fT4 levels were significantly lower in non-survivors, with respect to both 28-day mortality and in-hospital mortality. In contrast, no significant differences in TSH levels were observed between survivors and non-survivors. Similar results were reported by Rao et al. [27], with significantly lower fT3 and fT4 levels in non-survivors, but similar TSH levels.

Contrary to our findings, Wang et al. [15] reported no association between fT4 and in-hospital mortality, whereas total T4 was significantly lower among non-survivors. Sun et al. [18] identified a significant association between reduced levels of fT3, total T3, and the T3/fT3 ratio and mortality, while fT4, total T4, and TSH levels did not differ between survivors and non-survivors.

Most studies show a lack of correlation between TSH levels and mortality, in line with our results. However, in a study published in 2025, Wang et al. [1] reported that lower TSH levels, as well as impaired sensitivity to thyroid hormones, were linked to higher mortality.

While a growing body of evidence links ESS to increased mortality among critically ill patients, the fraction of thyroid hormones reported varies in different studies. Most studies have showed an association between low T3 and fT3 and poor outcomes, while evidence concerning fT4 and total T4 is variable [11,28]. This heterogeneity may reflect the dynamic nature of ESS, with a reduction in T3 and fT3 occurring early, followed by a decrease in T4 and fT4, during prolonged critical illness [11]. This may partially explain the high mortality in our study population, as most patients presented with low fT4 levels on ICU admission and were likely in more advanced phases of critical illness, due to significant chronic comorbidities and delayed hospital presentation.

The use of fT4 as a predictor of mortality in septic patients may be a simple and cost-effective strategy. Data regarding mortality among septic patients with ESS in Eastern Europe remains limited. To the best of our knowledge, only one study, conducted on 49 patients at a single hospital in Kraków, Poland, has reported data from this geographical area [17]. Our study is the first to investigate the predictive potential of fT4 for mortality among septic ICU patients in Romania. Recently, a small randomized controlled trial, conducted in Bosnia and Herzegovina, has been published, evaluating the role of T3 supplementation in ESS patients with septic shock [23].

In our univariate analysis, fT4 demonstrated a moderate predictive ability for 28-day mortality, while APACHE II and SOFA scores performed better. Similar results were shown for in-hospital mortality.

In a similar study conducted by Liu et al. [7], the AUC for fT3 and fT4 (0.92 and 0.89, respectively) were higher than that of the SOFA score (0.82) for 28-day mortality. The lower predictive performance of fT4 in our cohort may be attributable to the marked heterogeneity of our patient population, which included both medical and surgical patients, who were retrospectively analyzed over a period of seven years. In contrast, the study by Liu et al. included patients admitted within a single year, with data collected prospectively, thereby allowing for the selection of a more homogeneous cohort and minimizing potential confounding factors. Foks et al. [17] reported similar results, with an AUC for fT4 of 0.68 for 30-day mortality and 0.7 for in-hospital mortality. Wang et al. [15] reported an AUC of 0.65 for fT4 and mortality, and an AUC of 0.82 for a multiparametric model.

The combined use of multiple parameters for risk stratification showed an improved predictive performance. In our study, the multiparametric model had a good discriminative power for in-hospital mortality and a very good discriminative power for 28-day mortality. Variables included in the multivariate regression were selected based on their statistical significance in univariate analyses. Among severity scores, the SOFA score was favored over the simultaneous inclusion of SOFA and APACHE II, as these scores share overlapping components, and their combined use could reduce clinical relevance. Additionally, the SOFA score was preferred because it is included in the Sepsis-3 definition and offers greater practicality in routine clinical settings due to its simpler calculation.

Beyond patient risk stratification in sepsis, thyroid hormone levels may serve as tools to identify patients with ESS who could benefit from hormonal replacement therapy. Kovacevic et al. [23] reported that T3 supplementation was associated with improved survival in patients with low baseline T3 and T4, whereas T3 administration in patients with low T3 and normal T4 led to increased mortality.

The present study has several limitations. First, it is a retrospective observational study conducted on a relatively small cohort. The study population was heterogeneous, encompassing both patients with sepsis without shock at admission and patients with septic shock, as well as both medical and surgical cases. Although we included only patients with bacterial sepsis, excluding purely viral cases, we did not account for potential viral coinfections, which is particularly relevant given that some patients were admitted during the SARS-CoV-2 pandemic. The presence of viral coinfection, even in a small subset of patients, could have influenced mortality. Thyroid function was assessed upon clinical indication from the attending physician. A potential limitation could be the lack of a clear, systematic approach for selecting which patients would have fT4 and TSH measured, with possible inconsistencies between different physicians, and the relatively low proportion of patients where these parameters were available, out of all the septic patients admitted in the study period. Data was retrospectively retrieved from the patients’ files, and clinical scoring (SOFA and APACHE II) was performed by different physicians during routine clinical care. Therefore, one of the limitations of this study might be the variability between healthcare professionals. Additionally, hormone levels were only measured once, rather than being monitored dynamically. Given the complex and continuously evolving pathophysiological changes in sepsis, serial measurements could provide more insight. In a study conducted by Xu et al. [29], the maximum discriminative power of fT3 was observed on day 5 after admission, with an AUROC of 0.88, outperforming the SOFA score. Patients receiving amiodarone therapy (either chronically or during their ICU stay) were not excluded, despite its known inhibitory effects on deiodinase D1 activity and the peripheral conversion of T4 to T3 [9]. Those receiving heparin or glucocorticoids during the ICU admission were also not excluded, but this does not alter the results of the study, since thyroid function was assessed early in the course of critical illness. Since many patients had several comorbidities and organ dysfunctions, intraclass assessment of differences would mean significant overlap between subgroups. Thus, we could not evaluate differences in mortality and thyroid hormone profiles between subgroups. The variability in the combination of different comorbidities in our single-center cohort limits the generalizability of our results, and multi-center studies are required to confirm patients’ profile links to mortality.

Currently, there is substantial heterogeneity among studies evaluating the association between ESS and mortality in sepsis. Variability in patient inclusion criteria (sepsis vs. septic shock), type of infection (community- vs. hospital-acquired), underlying pathology (medical vs. surgical), and outcome definitions (28- or 30-day mortality vs. 90-day or in-hospital mortality) contribute to inconsistent findings. These methodological differences likely explain the discrepancies observed across studies regarding the predictive value of fT4 levels [8,30]. Further research might include thyroid hormone profiles, together with novel biomarkers, such as micro ARN (miR) miR-122 and miR-150 and plasma gelsolin (pGSN), or others, to better define sepsis phenotypes, which might guide clinicians in tailoring a personalized treatment approach for each patient [31].

## 5. Conclusions

Our research shows that decreased fT4 levels on ICU admission were independently associated with increased mortality in this cohort of septic patients. While fT4 levels showed moderate predictive ability for both 28-day and in-hospital mortality, TSH was similar between survivors and non-survivors. Given the significant heterogeneity in our study population, as well as between different reports in the literature, large prospective studies are warranted. Validation of multiparametric models, incorporating dynamic fT4 measurements, in subgroups defined by underlying pathology and geographic variability, may improve risk stratification and resource allocation. Moreover, the potential role of thyroid hormone supplementation in selected patients should be further investigated.

## Figures and Tables

**Table 1:** Comparison of demographic data, primary diagnosis and sources of infection between the study groups.

		28-Day Mortality			In-Hospital Mortality		
	All Patients (*n* = 149)	Survivors (*n* = 78)	Non-Survivors (*n* = 71)	*p*	Survivors (*n* = 59)	Non-Survivors (*n* = 90)	*p*
Sex, *n* (%)				0.247			0.216
M	64 (42.95)	37 (47.44)	27 (38.03)		29 (49.15)	35 (38.89)	
F	85 (57.05)	41 (52.56)	44 (61.97)		30 (50.85)	55 (61.11)	
Age (years), median (IQR)	73 (65; 80)	70.5 (61; 76.25)	75 (69; 82)	0.002	69 (57; 76)	74 (69; 82)	0.002
Primary diagnostic, *n* (%)				0.012			0.004
Sepsis	37 (24.83)	26 (33.33)	11 (15.49)		22 (37.29)	15 (16.67)	
Septic shock	112 (75.17)	52 (66.67)	60 (84.51)		37 (62.71)	75 (83.33)	
Location of primary infection, *n* (%)				0.977			0.422
One source	103 (69.13)	54 (69.23)	49 (69.01)		43 (72.88)	60 (66.67)	
Multiple sources	46 (30.87)	24 (30.77)	22 (30.99)		16 (27.12)	30 (33.33)	
Sepsis source ^1^, *n* (%)				N/A			N/A
Pulmonary	62 (41.61)	33 (42.31)	29 (40.85)		22 (37.29)	40 (44.44)	
Digestive	59 (39.6)	34 (43.59)	25 (35.21)		23 (38.98)	36 (40)	
Urinary	39 (26.17)	19 (24.36)	20 (28.17)		14 (23.73)	25 (27.78)	
Cutaneous	27 (18.12)	15 (19.23)	12 (16.9)		13 (22.03)	14 (15.56)	
Endocarditis	4 (2.68)	1 (1.28)	3 (4.23)		1 (1.69)	3 (3.33)	
Mediastinitis	1 (0.67)	0 (0)	1 (1.41)		0 (0)	1 (1.11)	
Septic arthritis	1 (0.67)	0 (0)	1 (1.41)		0 (0)	1 (1.11)	
Unknown	15 (10.07)	6 (7.69)	9 (12.68)		5 (8.47)	10 (11.11)	
Type of pathology, *n* (%)				0.011			0.147
Medical	89 (59.73)	39 (50)	50 (70.42)		31 (52.54)	58 (64.44)	
Surgical	60 (40.27)	39 (50)	21 (29.58)		28 (47.46)	32 (35.56)	
Hospital admission type, *n* (%)				0.622			0.999
Emergency admission	145 (97.32)	75 (96.15)	70 (98.59)		58 (98.31)	87 (96.67)	
Planned admission	4 (2.68)	3 (3.85)	1 (1.41)		1 (1.69)	3 (3.33)	

^1^ Sum of percentages is greater than 100 because some patients had multiple sources of sepsis.

**Table 2:** Comparison of patient’s comorbidities, critical illness severity scores and length of stay between the study groups.

		28-Day Mortality			In-Hospital Mortality		
	All Patients (*n* = 149)	Survivors (*n* = 78)	Non-Survivors (*n* = 71)	*p*	Survivors (*n* = 59)	Non-Survivors (*n* = 90)	*p*
HTN, *n* (%)	92 (61.74)	47 (60.26)	45 (63.38)	0.695	33 (55.93)	59 (65.56)	0.237
CHF, *n* (%)	84 (56.38)	38 (48.72)	46 (64.79)	0.048	25 (42.37)	59 (65.56)	0.005
AF, *n* (%)	56 (37.58)	26 (33.33)	30 (42.25)	0.262	18 (30.51)	38 (42.22)	0.149
DM, *n* (%)	51 (34.23)	29 (37.18)	22 (30.99)	0.426	20 (33.9)	31 (34.44)	0.945
CKD, *n* (%)	35 (23.49)	13 (16.67)	22 (30.99)	0.039	10 (16.95)	25 (27.78)	0.127
COPD, *n* (%)	31 (20.81)	11 (14.1)	20 (28.17)	0.035	10 (16.95)	21 (23.33)	0.348
Active or past cancer, *n* (%)	26 (17.45)	15 (19.23)	11 (15.49)	0.548	8 (13.56)	18 (20)	0.311
History of stroking, *n* (%)	24 (16.11)	12 (15.38)	12 (16.9)	0.801	6 (10.17)	18 (20)	0.11
Autoimmune disease, *n* (%)	11 (7.38)	4 (5.13)	7 (9.86)	0.27	2 (3.39)	9 (10)	0.201
APACHE II, median (IQR)	27 (20.75; 35)	22 (15; 32)	32 (26; 38.5)	<0.001	21 (14.5; 32)	31 (23.25; 38)	<0.001
SOFA, mean ± SD	11.18 ± 4.59	9.53 ± 4.73	12.97 ± 3.7	<0.001	9.17 ± 4.55	12.48 ± 4.14	<0.001
ICU LOS (days), median (IQR)	9 (5; 22)	11 (6; 29)	7 (4; 15.5)	0.006	7 (5; 12.5)	11 (5; 22.75)	0.209
Total hospital LOS (days), median (IQR)	22 (10; 35)	30.5 (17.25; 44.75)	13 (7; 25)	<0.001	23 (14; 34.5)	20 (8.25; 36)	0.208

Abbreviations: HTN, hypertension; CHF, congestive heart failure; AF, atrial fibrillation; DM, diabetes mellitus; CKD, chronic kidney disease; COPD, chronic obstructive pulmonary disease; APACHE II, Acute Physiology and Chronic Health Evaluation II; SOFA, Sequential Organ Failure Assessment (SOFA) Score; ICU, intensive care unit; LOS, length of stay.

**Table 3:** Comparison of patients’ laboratory variables between the study groups.

		28 Day-Mortality			In-Hospital Mortality		
	All Patients (*n* = 149)	Survivors (*n* = 78)	Non-Survivors (*n* = 71)	*p*	Survivors (*n* = 59)	Non-Survivors (*n* = 90)	*p*
CRP (mg/dL), median (IQR)	16.24 (7.26; 26.94)	17.21 (8.23; 32.03)	15 (6.72; 22.91)	0.234	17.19 (7.27; 24.56)	15.15 (7.27; 24.56)	0.349
PCT (ng/mL), median (IQR)	2.56 (0.78; 13.59)	1.59 (0.6; 14.25)	3.01 (1.03; 12.3)	0.241	1.57 (0.56; 13.57)	3.01 (0.98; 14.16)	0.124
WBC (×10^9^/L), median (IQR)	13.76 (9.46; 19.08)	12.61 (8.76; 18.16)	14.91 (10.68; 20.33)	0.221	12.01 (8.12; 17.35)	14.93 (10.81; 20.06)	0.018
Hemoglobin (g/dL), median (IQR)	10.6 (8.93; 12.05)	11.4 (9.2; 12.48)	9.75 (8.5; 11.4)	0.002	11.4 (9.4; 12.55)	9.8 (8.6; 11.6)	0.003
Platelet count (×10^9^/L), median (IQR)	232 (172.75; 308.75)	242.5 (181.75; 288.5)	215 (167; 309)	0.408	232 (177; 277.5)	238 (171.5; 313.5)	0.919
Serum creatinine (mg/dL), median (IQR)	1.59 (0.94; 3.18)	1.48 (0.8; 2.77)	1.96 (1.15; 3.49)	0.015	1.16 (0.73; 2)	2.09 (1.18; 3.89)	<0.001
TSH (μUI/mL), median (IQR)	2.01 (0.94; 3.36)	2.02 (0.98; 3.14)	2.01 (0.91; 3.56)	0.748	2 (1.02; 3.24)	2.05 (0.9; 3.44)	0.656
fT4 (ng/dL), mean ± SD	1.08 ± 0.35	1.15 ± 0.34	1 ± 0.35	0.01	1.16 ± 0.31	1.02 ± 0.37	0.014

Abbreviations: CRP, C-reactive protein; PLT, platelets; WBC, white blood cells; TSH, thyroid stimulating hormone; fT4, free thyroxine.

**Table 4:** Comparison of critical illness severity scores, length of stay and mortality between patients grouped by fT4 values.

	fT4 Level			*p*
	Hypothyroid (*n* = 50)	Euthyroid (*n* = 92)	Hyperthyroid (*n* = 7)	
APACHE II, median (IQR)	26 (21; 34)	29 (20; 35)	20 (14.5; 27.5)	0.325
SOFA, mean ± SD	10.71 ± 4.13	11.71 ± 4.73	7.57 ± 4.28	0.07
ICU LOS (days), median (IQR)	12 (5; 22.75)	8 (5; 17)	7 (5.5; 20)	0.59
Total hospital LOS (days), median (IQR)	22 (10.5; 34.75)	22.5 (9; 35.25)	19 (15.5; 28.5)	0.947
28-day mortality, *n* (%)	31 (62)	37 (40.22)	3 (42.86)	0.044
In-hospital mortality, *n* (%)	39 (78)	46 (50)	5 (71.43)	0.004

**Table 5:** Comparison of critical illness severity scores, length of stay and mortality between hypothyroid patients grouped by TSH levels.

	Hypothyroid Patients		*p*
	Concordant TSH (*n* = 43)	Discordant TSH (*n* = 7)	
APACHE II, median (IQR)	27.5 (21.25; 34.75)	26 (18.5; 28.5)	0.55
SOFA, mean ± SD	10.48 ± 4.03	12.14 ± 4.78	0.328
ICU LOS (days), median (IQR)	12 (5; 22.5)	20 (9; 25.5)	0.237
Total hospital LOS (days), median (IQR)	22 (12; 34)	20 (10; 39)	0.978
28-day mortality, *n* (%)	26 (60.47)	5 (71.43)	0.579
In-hospital mortality, *n* (%)	33 (76.74)	6 (85.71)	0.595

**Table 6:** Univariate analysis of potential predictors for primary and secondary outcomes.

	28 Day-Mortality			In-Hospital Mortality		
	OR (95% CI)	*p*	AUC	OR (95% CI)	*p*	AUC
Age (years)	1.05 (1.02–1.08)	0.003	0.65	1.05 (1.02–1.08)	0.002	0.655
Primary diagnostic (septic shock vs. sepsis)	2.73 (1.23–6.05)	0.014	0.308	2.97 (1.38–6.39)	0.005	0.342
CHF	1.94 (1.01–3.74)	0.049	0.423	2.59 (1.32–5.08)	0.006	0.477
APACHE II	1.08 (1.04–1.11)	<0.001	0.732	1.06 (1.03–1.10)	<0.001	0.697
SOFA	1.21 (1.11–1.32)	<0.001	0.727	1.20 (1.10–1.31)	<0.001	0.715
Hemoglobin (g/dL)	0.82 (0.71–0.95)	0.007	0.653	0.83 (0.71–0.96)	0.011	0.645
Serum creatinine (mg/dL)	1.19 (0.99–1.44)	0.066	0.617	1.37 (1.09–1.72)	0.006	0.674
fT4 (ng/dL)	0.28 (0.10–0.76)	0.012	0.615	0.3 (0.11–0.80)	0.017	0.632

**Table 7:** Multivariate models for prediction of primary and secondary outcomes.

	28-Day Mortality				In-Hospital Mortality			
	SE	OR (95% CI)	*p*	AUC (95% CI)	SE	OR (95% CI)	*p*	AUC (95% CI)
fT4 (ng/dL)	0.63	0.2 (0.06–0.69)	0.011	0.805 (0.733–0.876)	0.62	0.23 (0.07–0.78)	0.018	
Hb (g/dL)	0.08	0.82 (0.70–0.96)	0.014		0.08	0.83 (0.71–0.98)	0.029	0.799 (0.725–0.873)
Age (years)	0.02	1.06 (1.02–1.10)	0.005		0.02	1.05 (1.01–1.08)	0.008	
SOFA	0.05	1.22 (1.10–1.35)	<0.001		0.05	1.2 (1.09–1.32)	<0.001	

## Data Availability

Data are fully available from the corresponding author upon request, due to ethical aspects concerning patient privacy.
